# Zone 3 Aggressive Retinopathy of Prematurity in a Near-Term Delivered Big Baby With a Birth Weight of 3,200 g

**DOI:** 10.7759/cureus.104121

**Published:** 2026-02-23

**Authors:** K. Shreeya Jain, Brijesh Takkar, Akash Belenje

**Affiliations:** 1 Vitreoretina, Srimati Kanuri Santhamma Centre for Vitreo-Retinal Diseases, Anant Bajaj Retina Institute, Kallam Anji Reddy Campus, LV Prasad Eye Institute, Hyderabad, IND

**Keywords:** aggressive retinopathy of prematurity, a-rop, big baby a-rop, near-term baby a-rop, neonatal intensive care unit, zone 3 a-rop

## Abstract

Aggressive retinopathy of prematurity (A-ROP) represents a severe, rapidly progressive variant of retinopathy of prematurity (ROP) that is most observed in extremely premature, low-birth-weight infants and usually involves the posterior zones. Its occurrence in near-term infants with normal birth weight, involving Zone 3, is rare. This report dwells upon such a rare occurrence. This is a case of a male baby born at 37 weeks' gestation with a birth weight of 3,200 g. The baby required a neonatal ICU (NICU) admission for 13 days for respiratory distress and seizures. At a postmenstrual age of 40 weeks, fundus examination revealed A-ROP in both eyes in Zone 3. After parental counseling, bilateral intravitreal anti-vascular endothelial growth factor (anti-VEGF) therapy with bevacizumab (one-third adult dose, 0.4 mg/0.015 mL) was administered. The infant showed rapid regression of plus disease and hemorrhages.

Subsequent follow-ups demonstrated continued improvement, with complete retinal maturation by six months. At the final follow-up, the child had a mature retina, an unremarkable anterior segment, and a refraction of +0.50 DS/−2.00 DC × 180° in both eyes. This case highlights a rare presentation of A-ROP involving Zone 3 in a near-term, normal birth-weight infant. The potential role of postnatal factors, including respiratory distress management, in the development of A-ROP outside traditional risk profiles. The prognosis is good if the condition is treated promptly and there is no further systemic deterioration.

## Introduction

Retinopathy of prematurity (ROP) is a significant cause of visual morbidity and has remained a major contributor to childhood blindness globally since the mid-20th century. Globally, this condition is estimated to be responsible for at least 50,000 cases of childhood blindness every year [1]. The International Classification of Retinopathy of Prematurity (ICROP-3) has defined posterior Zone 2 as the retinal region within Zone 2, located two-disc diameters adjacent to the edge of Zone 1 [2]. In addition, it describes a continuum of vascular alterations from normal vasculature to plus disease, and it recommends the terminology “aggressive ROP” (A-ROP) rather than “aggressive posterior ROP” (APROP) [2]. APROP is defined as a severe ROP variant affecting Zone 1 or posterior Zone 2, with pronounced posterior pole vascular dilatation and tortuosity in all quadrants, flat extraretinal fibrovascular proliferation, and rapid disease progression [3]. Differences in clinical manifestations of ROP may be attributable to whether the insult affects vasculogenesis or angiogenesis. Vasculogenesis involves the de novo formation of retinal vessels from vascular precursor cells at the optic disc, whereas angiogenesis refers to vessel growth from existing vasculature [4]. Early disruption of vasculogenesis, particularly in extremely premature infants exposed to hyperoxia, may result in severe A-ROP confined to Zone 1 or Zone 2 [4].

Several independent risk factors have been associated with the development of A-ROP, including extreme prematurity, respiratory distress, anemia, low platelet counts, repeated infections or sepsis, intrauterine growth restriction, and chorioamnionitis [5-7]. Shah et al. documented A-ROP in preterm infants ≥28 weeks’ gestation and ≥1,000 g birth weight, highlighting its occurrence in larger babies and its association with unblended oxygen use [8]. We report a rare case of A-ROP in Zone 3 in a near-term baby with a birth weight of >3,000 g and low-risk demographics. The disruption of normal retinal vasculogenesis by a postnatal event like respiratory distress and seizures was a critical event, indicating the need for continued surveillance for Zone 3 maturation in bigger and near-term babies. 

## Case presentation

**Figure 1:**
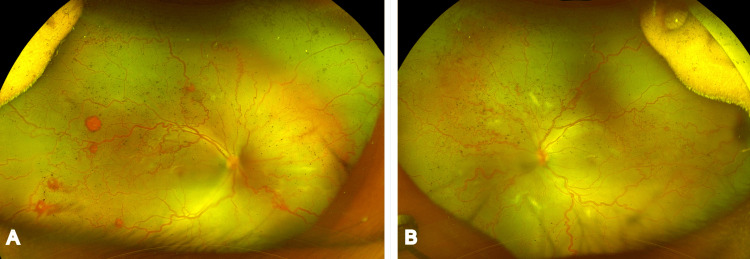
Ultra-widefield fundus image at presentation Figure 1A (right eye) and Figure 1B (left eye) show an ultra-widefield Optos (Optos Silverstone, Scotland) fundus image, showing A-ROP in Zone 3 with preretinal hemorrhages, nonphysiologic vascular loops, arteriovenous shunting, and marked vascular dilatation and tortuosity, without a demarcation line or ridge. There were a few Roth spots in the right eye. A-ROP: Aggressive retinopathy of prematurity

A male infant was delivered vaginally at 37 weeks of gestation, with a recorded birth weight of 3,200 g. At a postmenstrual age of 40 weeks, the baby was diagnosed with A-ROP and referred to our hospital for further management. The baby had a history of a neonatal ICU (NICU) stay for 13 days due to respiratory distress and seizures, with supplemental oxygen received during this period. At presentation, the anterior segments were normal with good pharmacologic dilation. Fundus evaluation revealed vessels reaching Zone 3 in both eyes with preretinal hemorrhages, nonphysiological vascular loops, arteriovenous shunting, and marked vascular dilatation and tortuosity bilaterally, without a demarcation line or ridge, and a few Roth spots in the right eye (Figure 1). The blood hemoglobin (Hb) of the baby was 10.4 g/dL (reference normal range of Hb is 11-17 g/dL).

**Figure 2:**
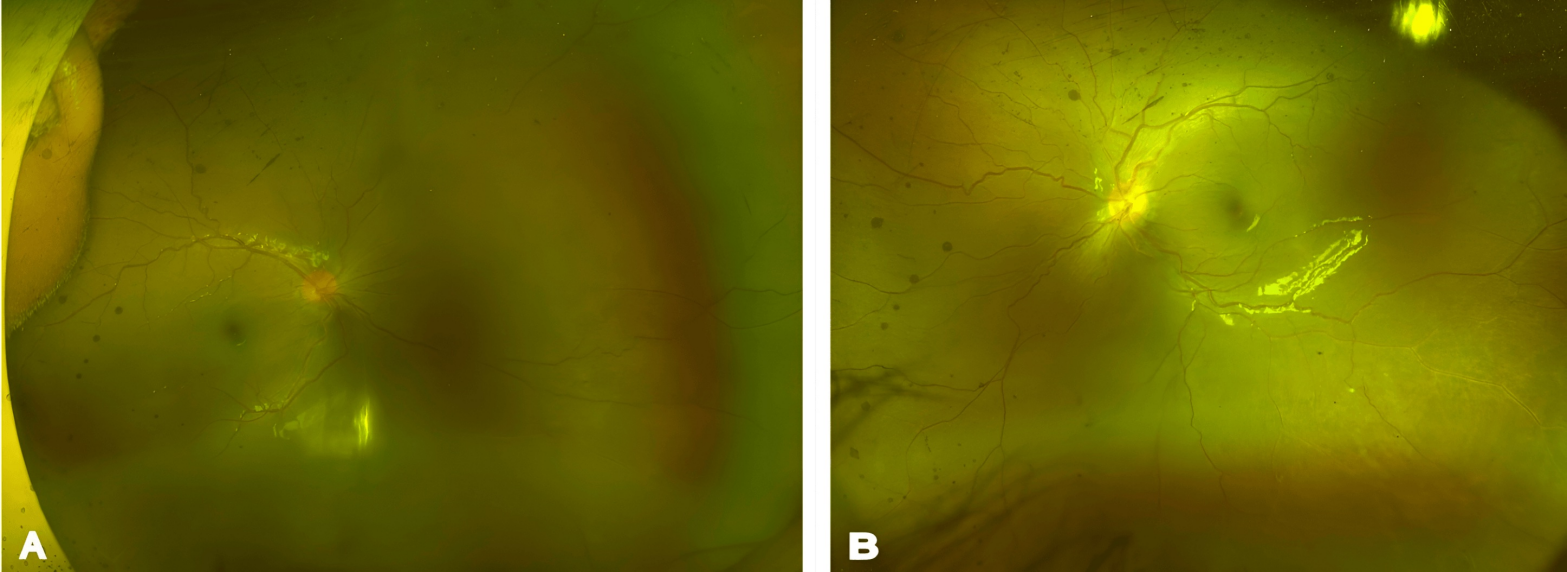
Ultra-widefield fundus image post treatment Figure 2A (right eye) and Figure 2B (left eye) show an ultra-widefield Optos fundus image at the sixth month follow-up. The baby had a mature retina in both eyes with no hemorrhages and vessels reaching the ora serrata.

Based on the clinical findings, a diagnosis of Zone 3 A-ROP was made in both eyes. After thorough counseling of the parents regarding the disease and treatment options, intravitreal anti-vascular endothelial growth factor (anti-VEGF) therapy - bevacizumab (one-third the adult dose, 0.4 mg/0.015mL) - was administered in both eyes. The baby was advised to undergo a complete blood count and encouraged to maintain Hb level above 10 g/dL. The baby was followed up once every three weeks and showed a reduction in plus disease with resolution of hemorrhages. At the sixth month follow-up, the baby had a mature retina with no hemorrhages and vessels reaching the ora serrata (Figure 2). Refraction in both eyes was +0.50 DS/-2.00 DC at 180°.

## Discussion

A-ROP carries a high risk of severe visual impairment if not managed promptly. Recognition of the importance of systematic ROP screening in vulnerable preterm infants is vital to prevent sight-threatening complications, including blindness. Several studies have explored the risk factors for A-ROP, formerly known as APROP, which has traditionally been reported in extremely premature and low-birth-weight infants [5,6]. Insults occurring during the vasculogenesis phase can disrupt normal vascular development, resulting in severe aggressive disease involving Zone 1. Such disturbances are more likely in infants with low gestational age, low birth weight and early exposure to oxygen supplementation [4]. The infant described in this report was born at 37 weeks’ gestation with a birth weight of 3,200 g, which is considerably higher than the traditionally recognized very low birth weight of less than 1,500 g for APROP. In a study by Sanghi et al., A-ROP occurring in heavier preterm infants weighing ≥1,500 g showed flat neovascularisation primarily involving posterior Zone 2, with atypical manifestations such as large vascular loops [7]. Our patient exhibited similar characteristics, with preretinal hemorrhages accompanied by dilated vessels and evolving vascular loops. Shah et al. reported the occurrence of APROP in 99 infants with a gestational age greater than 28 weeks and birth weight exceeding 1,000 g [8].

In our case, the baby was born with an appropriate birth weight, at a gestational age when retinal vascularisation of Zone 2 would be expected to be complete. However, we believe that following an abnormal hyperoxia exposure by oxygen supplementation for respiratory distress management at 37 weeks of gestation, the infant subsequently developed severe ROP, manifesting as A-ROP involving the anterior Zone 3. Moreover, the infant had a history of neonatal seizures, which were managed in the NICU.

The first epidemic of ROP resulted from unrestricted oxygen administration, while the second epidemic was driven by improved survival of very preterm infants in high-income countries. Currently, middle-income nations are experiencing the third epidemic of ROP, attributable to increased survival of preterm infants, variable quality of neonatal care, and limited coverage of ROP screening and treatment services [9]. The expansion of neonatal care services in middle-income countries has improved survival rates among preterm infants; however, the quality of care requires greater attention. In many middle-income countries, neonatal services have scaled up without parallel improvements in quality of care, infrastructure, and provider training. Consequently, wide variability in neonatal practices has been associated with the development of ROP in more mature and heavier infants. This shift increases the number of infants requiring screening and contributes to a higher burden of vision-threatening ROP [10,11]. Studies from India have demonstrated that ROP can develop in relatively mature and heavier infants, leading to revised screening guidelines that recommend examination of all infants with a birth weight <2,000 g or gestational age <34 weeks, as well as those with an unstable clinical course deemed at risk by a neonatologist or pediatrician [4,9]. To minimize variability and confusion regarding the timing of initial screening, Jalali et al., proposed that the first ROP examination be performed no later than within day 30 of life, and by day 20 of life in smaller infants (≤30 weeks’ gestation and/or birth weight ≤1,200 g) [12]. This “day 30/day 20” strategy facilitates adherence to screening timelines, as the date of birth is universally known and easily followed by healthcare providers [12]. Post intravitreal anti-VEGF in Zone 3 A-ROP, we can encounter reactivation and residual persistent avascular retina (PAR). Hence, it is important for continued surveillance for complete Zone 3 maturation in bigger and near-term babies.

## Conclusions

This case report describes an unusual occurrence of A-ROP in a near-term infant with a normal birth weight, presenting with severe disease involving Zone 3. Traditionally, A-ROP has been associated with extreme prematurity and low birth weight. The case report describes Zone 3 A-ROP in a near-term baby with a normal birth weight. The baby had a history of a neonatal intensive care stay for respiratory distress and seizures. This case report reiterates the potential role of postnatal factors, including respiratory management, in the development of severe ROP outside traditional risk profiles. This emphasizes the need for vigilance, timely screening, and management of bigger and near-term babies with a history of oxygen supplementation or NICU admission.
